# Climate‐Driven Range Dynamics of the Chinese Giant Salamander: Past, Present, and Future Projections From Ensemble Species Distribution Models

**DOI:** 10.1002/ece3.73474

**Published:** 2026-04-20

**Authors:** Chunlin Zhao, Zijian Sun, Yuanhong Shang, Zhong Xu, Yuanyong Gong, Lihua Zhao, Tao Li, Tian Zhao

**Affiliations:** ^1^ School of Biological and Chemical Engineering (School of Agriculture) Panzhihua University Panzhihua China; ^2^ College of Fisheries Southwest University Chongqing China; ^3^ Institute of Vanadium and Titanium Panzhihua University Panzhihua China

**Keywords:** *Andrias davidianus*, centroid shift, climate change, habitat suitability, species distribution models

## Abstract

Understanding how climate change impacts species distribution has become a central issue in conservation ecology and biodiversity management. As the largest extant amphibian species and a critically endangered vertebrate, the Chinese giant salamander ( *Andrias davidianus* ) serves as an important indicator for aquatic ecosystem health and climate vulnerability. In this study, we applied an ensemble species distribution modeling approach to reconstruct the historical distribution, assess the current habitat suitability, and project future range dynamics of *A. davidianus* across China. Key environmental drivers were identified, with the mean temperature of the coldest quarter (40.1%) being the most influential variable, followed by mean diurnal range (26.3%) and elevation (15.9%). Since the Mid‐Holocene, the total suitable habitat has expanded nearly 10‐fold, reaching 148.31 × 10^4^ km^2^ under current climatic conditions. By the 2090s, under the low‐emission scenario (SSP126), suitable habitats are projected to expand northwestward with a net areal increase, whereas under the high‐emission scenario (SSP585), habitats are likely to become increasingly fragmented and shift southeastward, accompanied by slight contraction. The distribution centroid has consistently migrated northeastward since the historical period and is expected to continue this trajectory in the future. Our findings provide critical insights into the climate‐driven habitat dynamics of *A. davidianus* and highlight the urgency of integrating climate adaptation into the conservation planning for this iconic endangered species.

## Graphical abstract

This study applies ensemble species distribution modeling to assess the climate‐driven range dynamics of the endangered Chinese giant salamander ( *Andrias davidianus* ). Results identify mean temperature of the coldest quarter as the primary environmental driver, reveal a nearly tenfold habitat expansion since the Mid‐Holocene, and project a continued northeastward range shift under future climate scenarios. These findings provide critical insights for conserving this iconic species under ongoing climate change.

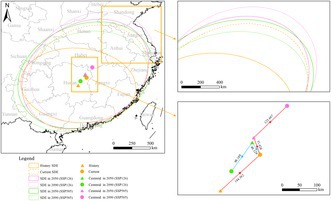

## 1 Introduction

Climate change has been considered to be one of the main factors that strongly affect animal species (Bellard et al. 2012; Mantyka‐Pringle et al. 2014). Evidence shows that these impacts extend across multiple biological levels, including species' phenology (Andreasson et al. 2023), behavior (Thompson and Hermann 2024), physiology, and population dynamics (Pecl et al. 2017; Radchuk et al. 2019). In recent decades, increasing studies have further revealed that climate change can trigger shifts in species' geographical distributions across both local and global scales (Jiang et al. 2023; Mi et al. 2023). For instance, at a local scale, Sun et al. (2025) projected a decline in overall habitat suitability for amphibians on Mount Emei under high‐emission scenarios, particularly in low‐altitude regions currently deemed highly suitable. On a broader scale, Beyer and Manica (2020) estimated that by 2100, the distribution ranges of 16,919 mammals, birds, and amphibian species could shrink by an average of 23%. Such range contractions often force species into smaller, fragmented habitats, which can reduce genetic diversity, limit resource availability, and increase extinction risk (Cahill et al. 2013; Román‐Palacios and Wiens 2020), ultimately impairing ecosystem functioning and biodiversity (Pecl et al. 2017). Therefore, elucidating how climate change influences species distribution is crucial for developing effective strategies for biodiversity conservation and management.

Amphibians are among the most threatened vertebrates globally (Stuart et al. 2004; Heatwole 2013). It is estimated that 49.7% of amphibian species worldwide will experience shifts in their geographic ranges because of climate change, whereas only 6.5% are likely to remain unaffected (Souza et al. 2023). This high vulnerability stems from their unique life history traits, such as dependence on both aquatic and terrestrial habitats, permeable skin, and limited dispersal ability, which collectively constrain their capacity to adapt to climatic shifts (Blaustein et al. 2003; Luedtke et al. 2023). In China, more than 60% of amphibian species have been identified as highly and moderately vulnerable to climate change (Zhao et al. 2022), underscoring the urgent need to prioritize amphibians in climate adaptation strategies. As one of the world's largest extant amphibians, the Chinese giant salamander ( *Andrias davidianus* ) has an evolutionary history dating back 16 million years (Fei et al. 2006; Yan et al. 2018). However, its wild populations have experienced severe declines caused by habitat degradation, overexploitation, water pollution, and genetic introgression (Liang et al. 2004; Yan et al. 2018; Zhao et al. 2020). As a result, the species is classified as Critically Endangered both globally (IUCN 2025) and within China (Jiang et al. 2016). Although several studies have used species distribution models (SDMs) to assess how climate change may influence the Chinese giant salamanders' suitable habitats (e.g., Chen et al. 2018; Zhang et al. 2020), these efforts have been constrained by limited occurrence records or coarse‐resolution climate data. More importantly, no study has yet systematically evaluated the distributional shifts of the Chinese giant salamander across historical, current, and future timeframes. Such a multi‐temporal perspective is essential, as understanding past species responses to climatic shifts can significantly improve future projections (Nogués‐Bravo 2009). Moreover, the morphological conservatism of cryptobranchids over evolutionary time renders giant salamanders a valuable group for environmental and paleoclimatic research (Böhme et al. 2012).

SDMs have become a widely used tool for assessing the impacts of climate change on species distribution patterns (Araújo et al. 2019). These statistical models correlate observed species occurrence data with environmental variables to infer ecological requirements and project habitat suitability (Austin 2002; Pearson et al. 2004). However, predictions on the basis of a single model algorithm often suffer from instability and substantial bias (Thuiller 2003; Araujo and New 2007). In contrast, modeling approaches integrate multiple algorithms to mitigate single‐model uncertainty and enhance predictive accuracy (Araujo and New 2007; Marmion et al. 2009). As a result, ensemble techniques are increasingly being adopted in ecological forecasting (e.g., Mateo et al. 2019; Hao et al. 2019).

In this study, we applied an ensemble species distribution modeling approach to assess the effects of climate change on the potential distribution of the Chinese giant salamander across China. This study advanced beyond previous SDM‐based assessments by explicitly integrating historical (Mid‐Holocene), contemporary, and future climate scenarios within a unified analytical framework, thereby offering a novel multi‐temporal perspective on distribution dynamics. Specifically, we first delineated the historical and contemporary suitable habitats of the species using a set of bioclimatic and environmental variables. This assessment allowed the evaluation of long‐term habitat stability and established a historical baseline. We then identified the key climatic drivers shaping its current distribution. Finally, we projected future habitat suitability under multiple climate scenarios and quantified shifts in distribution centroids to characterize range dynamics over time. These findings can improve our understanding of the spatiotemporal changes in suitable habitat for the Chinese giant salamander and provide a scientific foundation for its conservation and management under future climate conditions.

## 2 Materials and Methods

### 2.1 Study Area and Species Occurrence Records

**FIGURE 1:**
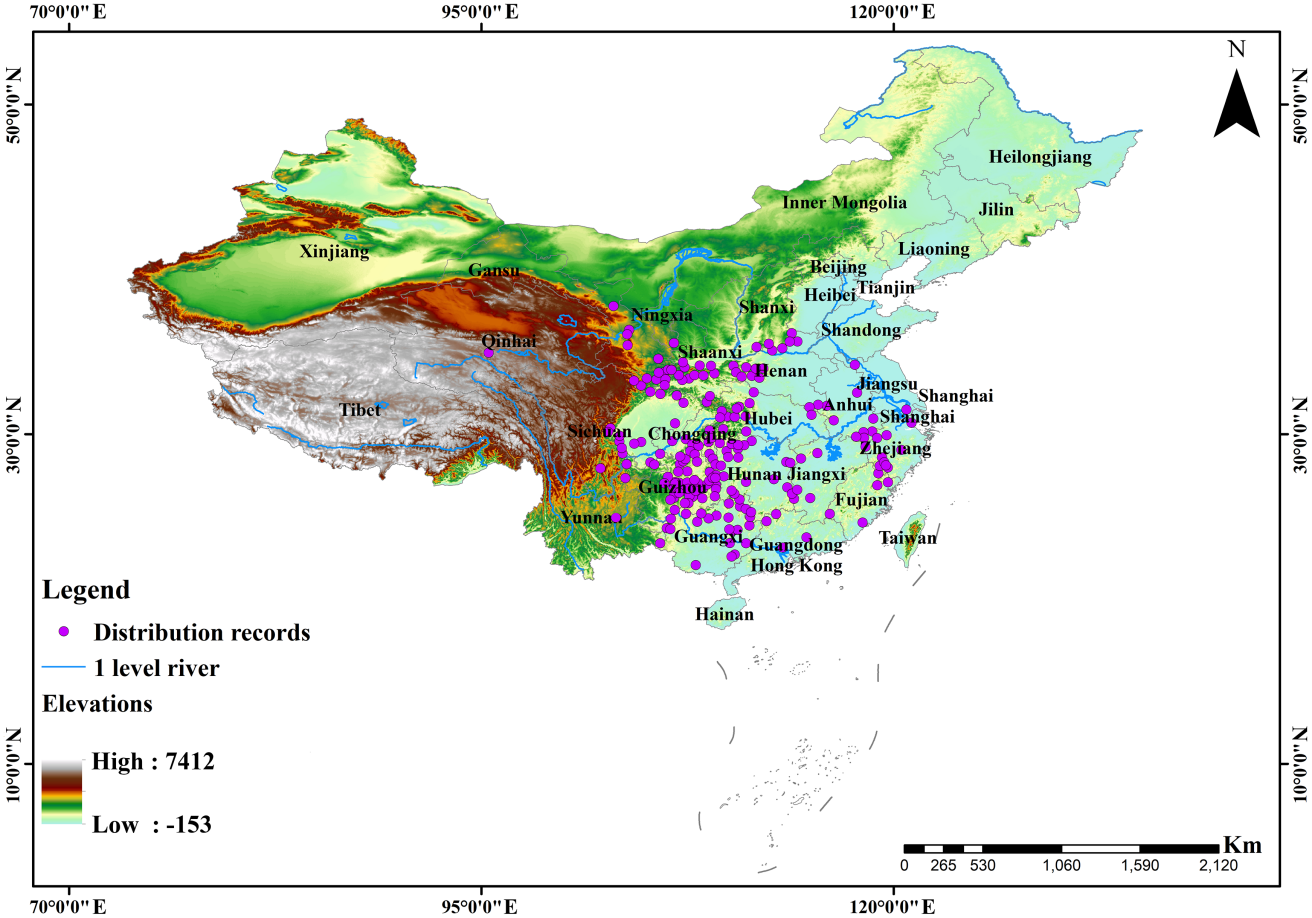
Spatial distribution of occurrence records used for modeling the habitat suitability of the Chinese giant salamander across China. A total of 213 unique presence points (purple) are shown, with one record retained per 2.5‐arcmin grid cell after spatial filtering to reduce autocorrelation.

The study encompassed the entirety of China, discretized into a 2.5′ × 2.5′ grid system. This spatial resolution (~5 km) was selected to match the resolution of the environmental predictors and is widely used in species distribution modeling to balance ecological relevance and spatial uncertainty (Hijmans et al. 2005; Elith and Leathwick 2009). This scope was selected as the Chinese giant salamander is historically known to be widely distributed across central and southern China (Fei et al. 2006; Chen et al. 2018; Turvey et al. 2019). Species occurrence records were compiled from published literature (Wang 2004; Fei et al. 2006, 2012; Shen et al. 2014; Yan et al. 2018; Turvey et al. 2019) and our recent field surveys. To ensure data quality, duplicate entries were removed using the “*dplyr*” and “*CoordinateCleaner*” packages in R (Zizka et al. 2019; Sun et al. 2025). Spatial autocorrelation was minimized by retaining only a single occurrence record per 2.5′ × 2.5′ grid cell. The final dataset comprised 213 unique records, each occupying a distinct grid cell (Figure 1).

### 2.2 Environmental Variables

On the basis of previous studies indicating that elevation, climate, and human population density are key determinants of the species' distribution (Turvey et al. 2019; Zhang et al. 2020), we initially considered 22 environmental variables across four major categories for modeling habitat suitability of the Chinese giant salamander (Table S1). Nineteen bioclimatic variables and elevation data at 2.5′ spatial resolution were sourced from WorldClim 2.1 (https://www.worldclim.org/; Fick and Hijmans 2017). Land cover class (1 km resolution) and vegetation data (500 m resolution) were obtained from global map data archives (https://globalmaps.github.io/). To ensure consistency, all bioclimatic variables (historical, current, and future) were acquired from WorldClim. Future climate projections under the SSP126 and SSP585 scenarios were represented by multi‐decadal averages for the 2050s (2041–2060) and 2090s (2081–2100), respectively, at the same spatial resolution as the current climate data. All variables were resampled to a uniform 2.5′ resolution. To mitigate multicollinearity, we performed variable selection on the basis of pairwise Pearson's correlation coefficients (*|r|* < 0.80), retaining only those variables with low redundancy for subsequent modeling (Zhao et al. 2021).

### 2.3 Modeling Procedure

We applied an ensemble species distribution modeling framework to project the historical, current, and future potential habitats of the Chinese giant salamander. Specifically, we employed 10 modeling algorithms, including artificial neural networks (ANN), classification tree analysis (CTA), flexible discriminant analysis (FDA), generalized additive model (GAM), generalized boosting model (GBM), generalized linear model (GLM), multiple adaptive regression splines (MARS), maximum entropy (MaxEnt), random forests (RF), and surface range envelope (SRE; Thuiller et al. 2019). For algorithms requiring absence data, we generated 10,000 pseudo‐absence points randomly across the study area, following established practices (Phillips et al. 2006; Merow et al. 2013; Guisan et al. 2017). Model performance was evaluated via four‐fold cross‐validation, with 75% of data used for training and the remainder for model testing (Guisan et al. 2017; Thuiller et al. 2019). Predictive accuracy was assessed using the true skill statistics (TSS) and the area under the receiver operating characteristic curve (AUC; Swets 1988; Allouche et al. 2006). Models with TSS > 0.70 were considered to exhibit good performance (Swets 1988; Allouche et al. 2006; Gallien et al. 2012). Predictor contributions were evaluated through randomization, and response curves were derived for the most influential variables. Predictions from individual algorithms were integrated using a committee averaging approach (Guisan et al. 2017; Thuiller et al. 2019). The resulting ensemble outputs were imported into ArcGIS 10.8, converted from ASC to raster format, and classified into four habitat suitability levels (i.e., high, moderate, low, and unsuitable habitat) using Jenks' natural breaks method (Tao et al. 2024). For historical reconstructions, which feature coarser resolution and simplified climate gradients, habitats were classified more broadly as suitable or unsuitable, consistent with a prior study (Raxworthy et al. 2003). Classification thresholds were set independently for each period to account for variation in environmental inputs and to better capture underlying data structures (Liu et al. 2005). The area and proportion of each suitability class were calculated for all periods, and trends in habitat change from current to future scenarios were assessed.

The standard deviational ellipse (SDE) was applied in ArcGIS v10.8 to quantitatively analyze potential range shifts and distribution centroid migration. This approach effectively quantifies directional trends and spatial dispersion of geographic features, providing a clear visualization of species distribution patterns (Wang et al. 2024). The centroid of each SDE was defined as the distribution center of the Chinese giant salamander for subsequent spatiotemporal analysis (Wang et al. 2024; Sun et al. 2025).

## 3 Results

### 3.1 Model Performance and Variable Contributions

Following multicollinearity analysis, nine environmental variables were retained to develop SDMs, including mean diurnal range, isothermality, temperature seasonality, temperature annual range, mean temperature of coldest quarter, annual precipitation, elevation, land cover class, and vegetation (Figure S1). All variables represented current climatic and environmental conditions, and subsequent modeling was performed accordingly. Among the 10 algorithms evaluated, eight (CTA, FDA, GAM, GBM, GLM, MARS, MAXENT, and RF) demonstrated superior predictive performance compared to ANN and SRE (Table 1) and were thus selected for ensemble model construction. The resulting ensemble model achieved means TSS and AUC values of 0.734 ± 0.038 and 0.903 ± 0.024, respectively, outperforming all individual algorithms. Variable importance analysis revealed that the mean temperature of the coldest quarter (40.1%) was the most influential factor shaping habitat suitability for the Chinese giant salamander, followed by mean diurnal range (26.3%) and elevation (15.9%; Table 2).

**TABLE 1:** Predictive performance of 10 species distribution modeling algorithms and the ensemble model for estimating current habitat suitability of the Chinese giant salamander.

Modeling algorithm	AUC	TSS
Artificial neural network (ANN)	0.878 ± 0.026	0.688 ± 0.047
Classification tree analysis (CTA)*	0.867 ± 0.024	0.709 ± 0.041
Flexible discriminant analysis (FDA)*	0.910 ± 0.018	0.740 ± 0.033
Generalized additive model (GAM)*	0.897 ± 0.018	0.730 ± 0.034
Generalized boosting model (GBM)*	0.914 ± 0.015	0.743 ± 0.033
Generalized linear model (GLM)*	0.919 ± 0.016	0.749 ± 0.041
Multiple adaptive regression splines (MARS)*	0.912 ± 0.015	0.750 ± 0.039
Maximum entropy (MAXENT)*	0.893 ± 0.021	0.718 ± 0.040
Random forest (RF)*	0.912 ± 0.016	0.736 ± 0.035
Surface range envelope (SRE)	0.824 ± 0.024	0.648 ± 0.049
Ensemble model	0.903 ± 0.024	0.734 ± 0.039

*Note:* Asterisks (*) denote algorithms with True Skill Statistic (TSS) values exceeding 0.70, indicating high predictive performance; these were selected for constructing the ensemble model. All values represent the mean ± standard deviation on the basis of four‐fold cross‐validation.

Abbreviations: AUC: Area Under the Receiver Operating Characteristic Curve; TSS: True Skill Statistic.

**TABLE 2:** Relative contributions of environmental predictors in the ensemble species distribution model for estimating current habitat suitability of the Chinese giant salamander.

Predictor variable	Contribution
Mean diurnal range (°C)	0.263 ± 0.004
Isothermality (Bio2/Bio7) (* 100)	0.009 ± 0.000
Temperature seasonality	0.097 ± 0.001
Temperature annual range (°C)	0.036 ± 0.001
Mean temperature of coldest quarter (°C)	0.401 ± 0.004
Annual precipitation (mm)	0.021 ± 0.000
Elevation	0.159 ± 0.002
Landcover class	0.014 ± 0.001
Vegetation	0.002 ± 0.000

*Note:* Data are presented as mean ± SD.

### 3.2 Historical Potential Suitable Habitat Distribution of the Chinese Giant Salamander

**FIGURE 2:**
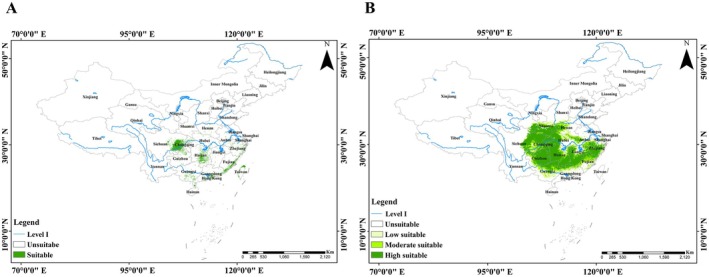
Predicted potential suitable habitats for the Chinese giant salamander during the Mid‐Holocene (A) and under current climatic conditions (B). Historical habitat suitability was classified into two categories (suitable vs. unavailable), whereas the current habitat was classified into four levels (high, moderate, low suitable, and unavailable). Major rivers (Level I) are shown for geographical reference.

Ensemble model projections indicate that during the Mid‐Holocene, the Chinese giant salamander's suitable habitat was primarily distributed across central and southern China. Core areas included western Chongqing, eastern and central Sichuan, central Hunan, southern Guangxi, Guangdong, southeastern Fujian, and Zhejiang, as well as western and northern Taiwan. Additionally, scattered suitable patches were identified in Shaanxi, Henan, Hubei, Jiangxi, Hainan, Yunnan, and Anhui provinces. The total suitable area was estimated at 14.99 × 10^4^ km^2^, representing 1.56% of the entire study area (Figure 2A).

### 3.3 Potentially Suitable Habitat for the Chinese Giant Salamander Under the Current Climate

Under current climatic conditions, the total area of suitable habitat for the Chinese giant salamander was 148.31 × 10^4^ km^2^, representing 15.45% of the study area. Highly suitable habitats were predominantly distributed across central and southeastern China, covering extensive portions of Sichuan, Chongqing, Hunan, Jiangxi, Hubei, Anhui, Zhejiang, Guizhou, Guangdong, Henan, Shaanxi, Fujian, and Guangxi provinces, with a total area of 96.21 × 10^4^ km^2^ (10.02% of the study area). Moderately suitable habitats were mainly identified in northern and southern regions, including parts of southern and western Anhui, central Jiangxi, Hubei, Guangxi, Shaanxi, and Fujian, southeastern Sichuan, southwestern Guizhou, western Chongqing, northeastern Hunan, northern Henan, and Guangdong, covering 29.58 × 10^4^ km^2^ (3.08% of the study area). Low suitability areas were primarily concentrated in central Shaanxi, Guangdong, northern Hubei, southern Shanxi, Guangxi, and Henan, spanning 22.52 × 10^4^ km^2^ (2.35% of the study area; Figure 2B).

### 3.4 Potential Suitable Habitat Distribution Under Future Climate Projections

**FIGURE 3:**
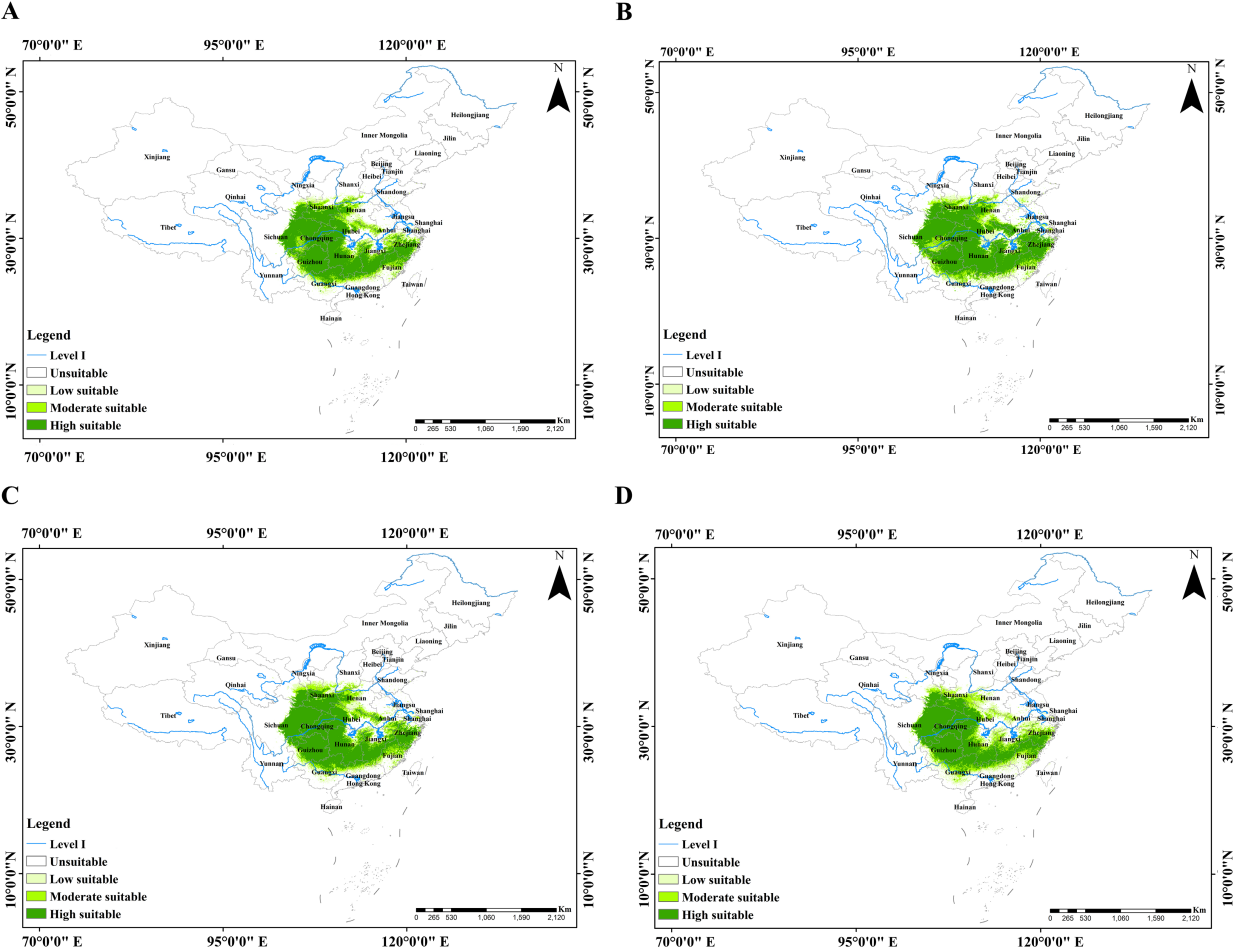
Projected potential suitable habitat distribution for the Chinese giant salamander under future climate scenarios. (A,B) The 2050s and 2090s under SSP126. (C,D) The 2050s and 2090s under SSP585. Habitat suitability is classified into four levels: High, moderate, low suitable, and unsuitable. Major rivers (Level I) are shown for reference.

Model projections for the 2050s indicate that under both SSP126 and SSP585 scenarios, suitable habitats for the Chinese giant salamander remain concentrated in central and southeastern China. Regions such as eastern Sichuan, southern Shaanxi, Chongqing, and adjacent provinces continue to support highly suitable habitats (Figure 3A,C). Relative to the current distribution, the total suitable area shows a slight contraction of 0.19% under SSP126, but a marginal expansion of 0.61% under SSP585 (Table S2).

By the 2090s, under the SSP126 scenario, suitable habitats are projected to expand northward and westward, particularly into southern Gansu, southern Shanxi, and northeastern Sichuan, resulting in a net increase of 1.14% in suitable area (Figure 3B). In contrast, under SSP585, suitable habitats become more fragmented and shift predominantly southward and eastward, particularly toward southern Zhejiang and northern Fujian, accompanied by a slight contraction of 0.32% in total area (Figure 3D; Table S2).

Overall, future changes in habitat suitability are characterized by increases in northern Shaanxi, Chongqing, and eastern Sichuan, alongside decreases in northern Zhejiang. A general northwestward shift toward high‐altitude regions is evident, suggesting that central China is likely to emerge as a critical high‐suitability zone under future climate scenarios (Figure 3).

### 3.5 Spatiotemporal Dynamics of Suitable Habitats

**FIGURE 4:**
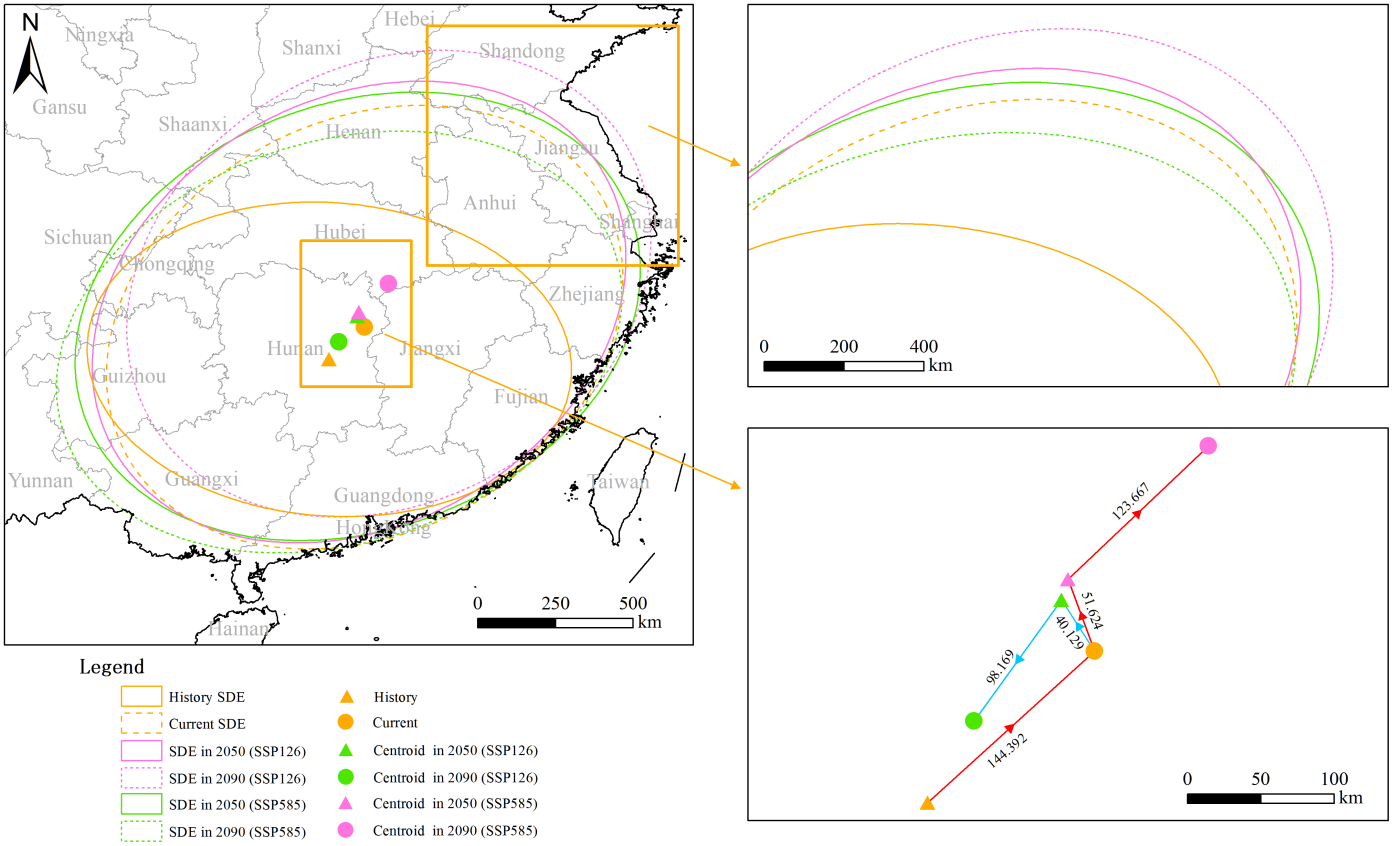
Spatiotemporal shifts in the distribution range and centroid of the Chinese giant salamander under historical, current, and future climate scenarios. The standard deviational ellipses (SDEs) illustrate the directional trends and dispersion of suitable habitats, whereas the centroids track the geographical center of distribution across periods. Arrows indicate the trajectory of centroid migration from historical to future conditions under SSP126 and SSP585 scenarios.

Analysis of distribution centroid migration reveals a northeastward shift of approximately 144.392 km in the Chinese giant salamander's range from the historical period to the present (Figure 4, Table S3). Under future climate scenarios, this northeastward trajectory continues but with varying magnitudes. In the SSP126 scenario, the centroid is projected to shift 40.129 km by the 2050s, followed by an additional 98.169 km by the 2090s, resulting in a final position west of the 2050s centroid. A similar pattern emerges under SSP585, with displacements of 51.624 km by the 2050s and 123.667 km by the 2090s, indicating greater overall movement under the high‐emission scenario (Figure 4, Table S3).

## 4 Discussion

This study systematically elucidates the spatiotemporal dynamics of habitat suitability for the Chinese giant salamander and identifies its key climatic drivers. Our results demonstrate that the mean temperature of the coldest quarter is the primary environmental factor constraining the species' distribution, reflecting its high sensitivity to extreme winter conditions. Notably, the current suitable habitat (148.31 × 10^4^ km^2^) represents an estimated approximately tenfold expansion since the Mid‐Holocene, suggesting that historical global warming may have facilitated range enlargement for this species. However, the magnitude of this expansion may be interpreted cautiously, as it is subject to uncertainties inherent in paleoclimate reconstructions and paleo‐species distribution modeling approaches (Varela et al. 2011; Huntley 2012). This historical range expansion contrasts with the projected habitat fragmentation under SSP585. This difference likely reflects contrasts in the rate and complexity of climatic change between past and future periods: Mid‐Holocene warming occurred more gradually, whereas future warming is expected to be faster and accompanied by greater climatic variability and extremes, which can disrupt aquatic habitat continuity and promote fragmentation (Chen et al. 2011a; Pecl et al. 2017; Pottier et al. 2025). In addition, anthropogenic pressures such as dam construction, water pollution, and river modification, which were largely absent during the Holocene, are now pervasive and can interact synergistically with climate change to further fragment habitats and impede dispersal, thereby exacerbating the differences between past and projected range dynamics. Under future climate scenarios (SSP126/SSP585), suitable habitats are projected to shift continuously northwestward toward higher elevations, with central China consolidating as a core high‐suitability region. This migratory trajectory appears to be driven by intensified temperature seasonality and altered precipitation patterns, underscoring how climate change can reshape species distributions by modifying temperature‐precipitation interactions (Ma et al. 2024). Collectively, these findings provide important insights into the response mechanisms of endangered amphibians to ongoing climate change.

### 4.1 Important Predictors and Model Accuracy

The appropriate selection of predictor variables is critical for reliable species distribution modeling (Synes and Osborne 2011; Harris et al. 2013; Fourcade et al. 2018). Following multicollinearity screening, nine variables were retained for modeling the distribution of the Chinese giant salamander. Among these, the mean temperature of the coldest quarter and the mean diurnal range showed the strongest associations with the species' occurrence. These findings align with previous studies, reporting that the species prefers a temperate range of 5°C–25°C and humid conditions, while being particularly vulnerable to temperature extremes (Zhang et al. 2006, 2014; Mu et al. 2011; Zhu et al. 2022). Temperature is widely recognized as a key determinant of species distributions across broad spatial scales (Guo et al. 2021), and the inclusion of these biologically relevant predictors contributed to the high predictive accuracy of our ensemble model (i.e., TSS > 0.70; Zhang et al. 2020).

### 4.2 History Potential Suitable Habitat for the Chinese Giant Salamander

Our reconstructions suggest that during the Mid‐Holocene, the Chinese giant salamander exhibited a patchy distribution, primarily confined to low‐elevation landscapes such as the middle and lower Yangtze River basin and adjacent coastal zones. This distribution pattern corresponds to the species' known preference for dense vegetation cover (Guo 2011), which was supported by the extensive tropical evergreen broadleaf forests prevailing in these regions at the time (Song 1988). Paleoclimate reconstructions further indicate that the Mid‐Holocene climate in China was characterized by higher mean annual temperatures and greater climatic complexity than present conditions (Wu et al. 2017; Chen et al. 2020; Zhang et al. 2020). These warmer and more humid conditions may have limited the availability of suitable aquatic refugia, contributing to reduced population sizes and a fragmented species range. As the Mid‐Holocene represents an important climatic analog for projected future warming and humidity increases, these historical insights provide a valuable perspective for predicting and managing the habitat of the Chinese giant salamander under ongoing climate change (Zhang et al. 2020).

### 4.3 Potentially Suitable Habitat Under Current Climatic Conditions

The projected suitable habitat for the Chinese giant salamander aligns well with previously documented distributions (Chen et al. 2018; Zhang et al. 2020), primarily encompassing mid‐subtropical provinces in central and southeastern China. Key regions include Sichuan, Chongqing, Hunan, Jiangxi, Hubei, Anhui, Zhejiang, Guizhou, Guangdong, and Guangxi. This distribution pattern can be attributed to the region's moderate temperatures (falling within the species' optimal range of 5°C–25°C), coupled with abundant rainfall and a dense river network that provides essential aquatic habitats for these fully aquatic amphibians. These areas also support rich food resources, ensuring a stable trophic supply. Consequently, these provinces constitute the current ecological stronghold for the species and should be designated as conservation priorities.

### 4.4 Future Potential Suitable Habitat for the Chinese Giant Salamander

Our projections reveal a clear trend of suitable shifting toward higher altitudes and latitudes under future climate scenarios. Habitat suitability is expected to increase substantially in regions such as southern Shaanxi, Chongqing, Guizhou, and eastern Sichuan, attributable to relatively stable thermal regimes and favorable hydrological conditions (You et al. 2002; B. Wang 2020). In contrast, suitability is projected to decline in parts of southern Zhejiang and southwestern Hunan, indicating reduced climatic suitability in these current distribution areas. These shifts are amplified under high‐emission scenarios, with notable habitat contraction predicted in low‐altitude and low‐latitude regions. The observed distributional changes align with broader patterns of climate‐driven range shifts in amphibians, where species increasingly move poleward or upward in elevation to track suitable thermal environments (Chen et al. 2011b; Chen et al. 2011a; Zhao et al. 2017; Zhang et al. 2020; Shin et al. 2021). Enhanced surface runoff and moisture availability in southwestern China, particularly in karst landscapes such as Guizhou, may further improve habitat suitability under future climate conditions (You et al. 2002; B. Wang 2020). Our findings are consistent with previous studies reporting northward and upward shifts in amphibian species under climate change (Popescu et al. 2013; Duan et al. 2016; Zhang et al. 2020). However, the magnitude and direction of projected shifts can vary depending on the dispersal assumptions incorporated in species distribution models. Studies comparing unlimited versus no‐dispersal scenarios often show expanded ranges under unlimited dispersal but significant contractions when dispersal is restricted (Araújo et al. 2006; Li et al. 2013; Guisan et al. 2017). In this study, we assumed unlimited dispersal, which revealed a mosaic of range expansions and contractions across different provinces. Whether the Chinese giant salamander can successfully colonize newly suitable habitats remains uncertain and merits further study. Nonetheless, under all dispersal scenarios, our results consistently underscore the species' high vulnerability to ongoing climate change (Zhang et al. 2020).

### 4.5 Climate‐Driven Shifts in Distribution Centroid and Conservation Implications

Our findings demonstrated a consistent northeastward shift in the distribution centroid of the Chinese giant salamander under future climate scenarios, with more pronounced displacement under the high‐emission pathway (SSP585) than under the low‐emission scenario (SSP126). This directional movement aligns with the general expectation that species will shift their ranges toward higher latitudes or cooler regions in response to warming (Chen et al. 2011a; Pecl et al. 2017). However, meta‐analyses indicate that fewer than half of documented range shifts follow such “expected” trajectories, underscoring the substantial variation in species‐ and context‐specific responses (Rubenstein et al. 2023). The northeastward migration observed here likely reflects the combined influence of climatic and elevational gradients across China and highlights the importance of incorporating region‐specific dispersal capacity and habitat connectivity into range shift projections. Amphibians, such as the Chinese giant salamander, with their limited dispersal ability and strong reliance on aquatic habitats, may be particularly vulnerable to lagging behind the pace of climate change (Pottier et al. 2025). Therefore, although the centroid shifts detected in this study suggest a capacity for climate‐driven redistribution, effective conservation strategies must also address movement barriers and evolving habitat structures.

To enhance climate resilience, conservation efforts should prioritize maintaining and restoring hydrological connectivity among fragmented habitats, particularly by protecting river corridor networks and riparian buffer zones. Specifically, establishing ecological corridors that link currently occupied areas in Sichuan, Chongqing, and Hunan with projected future suitable regions in southern Shaanxi and Guizhou could facilitate natural dispersal and maintain genetic exchange (e.g., along the upper Yangtze and its tributaries). Additionally, assisted rewilding or reintroduction could be considered in areas identified as stable long‐term refugia under both climate scenarios, such as the mountainous interfaces of Sichuan, Chongqing, and Guizhou, provided that habitat quality, disease risks, and genetic compatibility are carefully evaluated prior to any translocation. For regions where habitat suitability is projected to decline (e.g., parts of Zhejiang and Hunan), in situ protection may need to be complemented by ex situ conservation measures.

This study also has several limitations. First, although the ensemble modeling approach improved prediction reliability, inherent uncertainties in species distribution models persist, particularly those associated with future climate projections and dispersal assumptions. Second, our models primarily incorporated climatic and topographic variables; other biologically relevant factors, such as human disturbance, biotic interactions, and habitat fragmentation, also strongly influence the distribution of the Chinese giant salamander but were not explicitly included. Anthropogenic factors such as dam construction, water pollution, and river channel modification may interact with climate change to further reduce habitat suitability. Dams can disrupt hydrological connectivity and restrict dispersal pathways, limiting the species' ability to track climate‐driven habitat shifts. Meanwhile, water pollution and altered flow regimes may degrade aquatic habitat quality and intensify physiological stress under warming conditions. Together, these pressures may amplify habitat fragmentation and population isolation beyond climate effects alone (Dudgeon et al. 2006; Reid et al. 2019). Consequently, the exclusion of these anthropogenic disturbance factors may lead to overestimation of habitat suitability, particularly in densely populated or heavily modified river basins where climatically suitable areas may not correspond to viable habitats. Therefore, caution is required when interpreting climatically suitable regions as indicators of potential species persistence. To improve future modeling efforts for this and other aquatic species, we recommend explicitly incorporating hydrological variables, such as stream flow dynamics, water temperature, and water quality parameters, which directly regulate habitat suitability and population persistence in riverine amphibians. Integrating such predictors may enhance the ecological realism and predictive accuracy of species distribution models under climate change (Dudgeon et al. 2006; Elith and Leathwick 2009). Despite these limitations, this research offers valuable insights by integrating multiple modeling techniques and evaluating habitat suitability across historical, current, and future timeframes. The comprehensive spatiotemporal assessment provided here can help inform targeted conservation strategies for this endangered amphibian in a changing climate.

## 5 Conclusions

In conclusion, this study provides an integrated assessment of climate‐driven distribution dynamics and key environmental determinants of the Chinese giant salamander using an ensemble species distribution modeling framework. Our results identify the mean temperature of the coldest quarter, mean diurnal range, and elevation as the predominant environmental factors shaping the species' distribution. Since the Mid‐Holocene, suitable habitats appear to have expanded substantially and are projected to continue shifting northwestward toward higher altitudes and latitudes, indicating that central and southwestern China are likely to function as critical long‐term climatic refugia. Concurrently, the species' distribution centroid is projected to shift northeastward under future warming, with more pronounced shifts under high‐emission scenarios, highlighting the importance of incorporating climate‐induced range dynamics into conservation frameworks.

These findings highlight the need to integrate climate change projections into conservation planning for this endangered species. Delineating future refugia and areas of habitat contraction may provide a basis for prioritizing measures such as habitat protection, restoration, and potential assisted migration. Conservation strategies should consider emphasizing the maintenance of habitat connectivity and microhabitat heterogeneity within projected high‐suitability zones. Future research would benefit from integrating physiological, behavioral, and genomic data to improve predictive accuracy while also accounting for realistic dispersal constraints and landscape fragmentation effects. Finally, implementing long‐term population monitoring and viability analyses will likely be important for developing adaptive management strategies capable of addressing the ongoing challenges posed by climate change.

## Author Contributions

**Chunlin Zhao:** data curation (equal), formal analysis (equal), methodology (equal), visualization (equal), writing – original draft (lead), writing – review and editing (supporting). **Zijian Sun:** investigation (equal), writing – original draft (supporting), writing – review and editing (supporting). **Yuanhong Shang:** formal analysis (equal), investigation (equal), supervision (equal), writing – original draft (supporting), writing – review and editing (supporting). **Zhong Xu:** conceptualization (equal), formal analysis (supporting), supervision (equal), writing – review and editing (supporting). **Yuanyong Gong:** conceptualization (equal), data curation (equal), project administration (equal), writing – original draft (supporting), writing – review and editing (supporting). **Lihua Zhao:** conceptualization (equal), data curation (equal), project administration (equal), writing – original draft (supporting), writing – review and editing (equal). **Tao Li:** conceptualization (equal), data curation (equal), project administration (equal), writing – original draft (supporting), writing – review and editing (equal). **Tian Zhao:** data curation (equal), formal analysis (equal), investigation (equal), methodology (equal), project administration (equal), visualization (equal), writing – original draft (lead), writing – review and editing (equal).

## Funding

This work was supported by the National Natural Science Foundation of China (grant number: 32370553), the Zhilan Foundation (grant number: 2024100501B), the Panzhihua Municipal‐level Guiding Science and Technology Plan Project (grant number: 2024ZD‐N‐1), and China Biodiversity Observation Networks (Sino BON).

## Conflicts of Interest

The authors declare no conflicts of interest.

## Supporting information


**Table S1:** Twenty‐two environmental variables were used in this study.
**Table S2:** Changes in the distribution of suitable and unsuitable habitats for the Chinese giant salamander under current and future climate scenarios (SSP126 and SSP585) in the 2050s and 2090s.
**Table S3:** Geographic coordinates of the standard deviational ellipse (SDE) centroid for the Chinese giant salamander under historical, current, and future climate scenarios.
**Figure S1:** Correlation matrix of the 22 environmental predictor variables considered in the species distribution modeling. Variables enclosed in red boxes indicate pairs with high collinearity, defined as an absolute Pearson's correlation coefficient |*r*| > 0.80. The nine variables retained for final modeling are those outside of the red boxes and not involved in strong collinearity.

## Data Availability

The species occurrence data supporting this study will be publicly available upon publication via the Figshare repository: https://doi.org/10.6084/m9.figshare.31604245.
