# Correction to “Climate‐Driven Range Dynamics of the Chinese Giant Salamander: Past, Present, and Future Projections From Ensemble Species Distribution Models”

**DOI:** 10.1002/ece3.74161

**Published:** 2026-08-06

Zhao, C. , Z. Sun , Y. Shang , et al. 2026. “Climate‐Driven Range Dynamics of the Chinese Giant Salamander: Past, Present, and Future Projections From Ensemble Species Distribution Models.” Ecology and Evolution 16, no. 4: e73474. 10.1002/ece3.73474.42017119 PMC13093209

The Equal Contribution statement was missing from the first‐page footnote. It should read: “Zijian Sun and Yuanhong Shang contributed equally to this work and share first authorship.”

We apologize for this error.

