# Supplementary material for: Climate‐Driven Range Dynamics of the Chinese Giant Salamander: Past, Present, and Future Projections From Ensemble Species Distribution Models
Source: Ecol Evol. 2026 Apr 20;16(4):e73474. doi: 10.1002/ece3.73474 (PMC13093209; doi:10.1002/ece3.73474)
Supplement: Supplementary file 1 — Table S1: Twenty‐two environmental variables were used in this study. Table S2: Changes in the distribution of suitable and unsuitable habitats for the Chinese giant salamander under current and future climate scenarios (SSP126 and SSP585) in the 2050s and 2090s. Table S3: Geographic coordinates of the standard deviational ellipse (SDE) centroid for the Chinese giant salamander under historical, current, and future climate scenarios. Figure S1: Correlation matrix of the 22 environmental predictor variables considered in the species distribution modeling. Variables enclosed in red boxes indicate pairs with high collinearity, defined as an absolute Pearson's correlation coefficient |r| > 0.80. The nine variables retained for final modeling are those outside of the red boxes and not involved in strong collinearity. [file ECE3-16-e73474-s001.docx]

**Table S1.** Twenty-two environmental variables were used in this study.

| **Category** | **Variable** | **Description** | **Unit** |
| --- | --- | --- | --- |
| Temperature-related Variables | Bio1 | Annual mean temperature | °C |
|  | Bio2 | Mean diurnal range | °C |
|  | Bio3 | Isothermality (Bio2/Bio7) (* 100) | NA |
|  | Bio4 | Temperature seasonality | NA |
|  | Bio5 | Max temperature of warmest month | °C |
|  | Bio6 | Min temperature of coldest month | °C |
|  | Bio7 | Temperature annual range | °C |
|  | Bio8 | Mean temperature of wettest quarter | °C |
|  | Bio9 | Mean temperature of driest quarter | °C |
|  | Bio10 | Mean temperature of warmest quarter | °C |
|  | Bio11 | Mean temperature of coldest quarter | °C |
| Precipitation-related Variables | Bio12 | Annual precipitation | mm |
|  | Bio13 | Precipitation of wettest month | mm |
|  | Bio14 | Precipitation of driest month | mm |
|  | Bio15 | Precipitation seasonality | mm |
|  | Bio16 | Precipitation of wettest quarter | mm |
|  | Bio17 | Precipitation of driest quarter | mm |
|  | Bio18 | Precipitation of warmest quarter | mm |
|  | Bio19 | Precipitation of coldest quarter | mm |
| Topographic Variable | Elevation |  | m |
| Vegetation and Landcover Variables | Landcover class |  | NA |
|  | Vegetation |  | NA |

**Table S2.** Changes in the distribution of suitable and unsuitable habitats for the Chinese giant salamander under current and future climate scenarios (SSP126 and SSP585) in the 2050s and 2090s.

| **Year/**  **Scenarion** | **Unsuitable habitat (km^2^)** | **Unsuitable Habitat (%)** | **Suitable habitat (km^2^)** | **Suitable Habitat (%)** | **Compare to current suitable habitat change (%)** |
| --- | --- | --- | --- | --- | --- |
| Current | 811.69 | 84.55 | 148.61 | 15.45 |  |
| 2050 |  |  |  |  |  |
| SSP126 | 813.52 | 84.74 | 146.48 | 15.26 | -0.19 |
| SSP585 | 805.81 | 83.94 | 154.19 | 16.06 | 0.61 |
| 2090 |  |  |  |  |  |
| SSP126 | 800.78 | 83.41 | 159.22 | 16.59 | 1.14 |
| SSP585 | 814.75 | 84.87 | 145.25 | 15.13 | -0.32 |

**Table S3.** Geographic coordinates of the standard deviational ellipse (SDE) centroid for the Chinese giant salamander under historical, current, and future climate scenarios.

| **Centroid** | **Longitude** | **Latitude** |
| --- | --- | --- |
| History | 112.493815 | 27.291656 |
| Current | 113.518514 | 28.222346 |
| 2050 under SSP126 | 113.356503 | 28.662082 |
| 2090 under SSP126 | 114.218143 | 29.480444 |
| 2050 under SSP585 | 113.316743 | 28.535536 |
| 2090 under SSP585 | 112.780047 | 27.791917 |


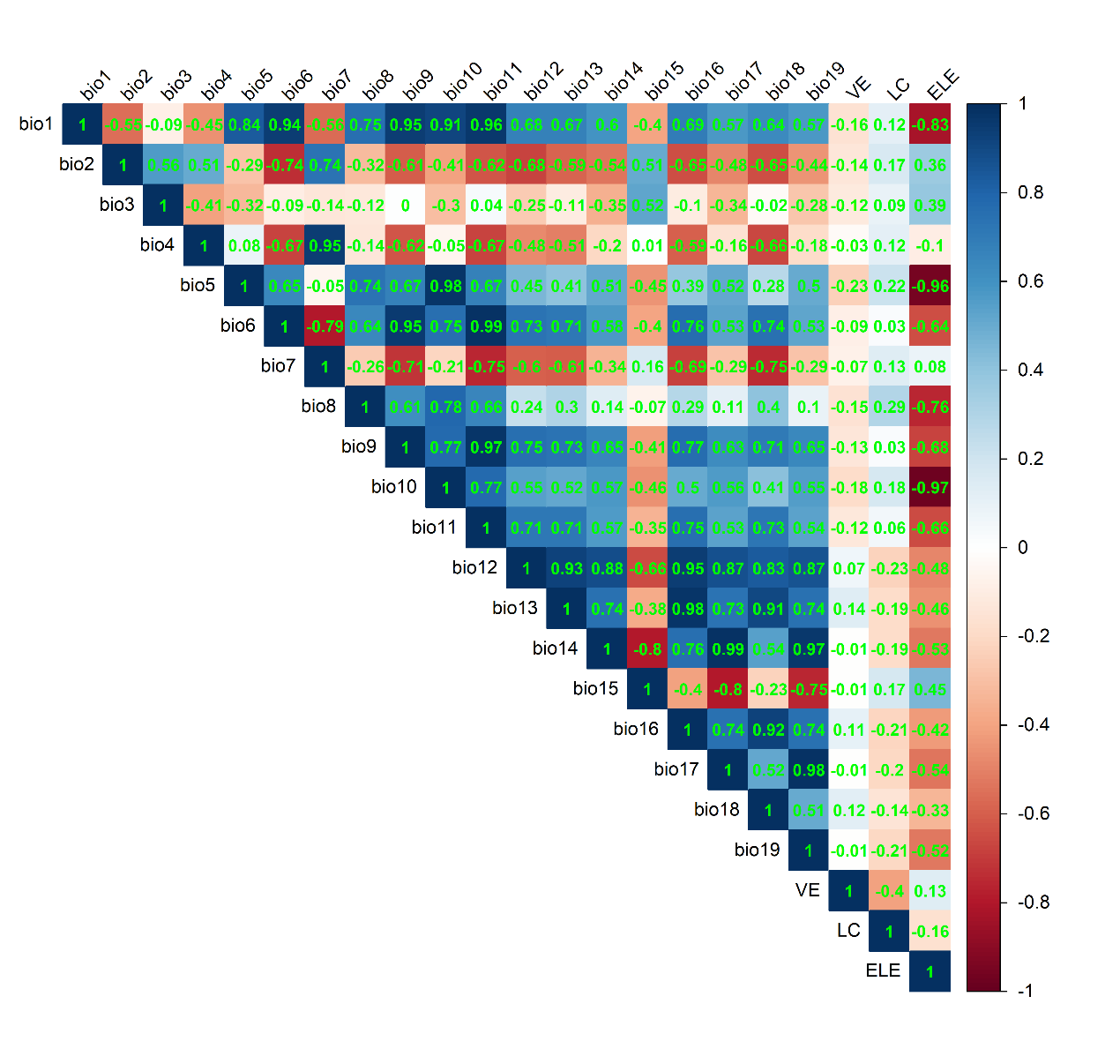


**Figure S1.** Correlation matrix of the 22 environmental predictor variables considered in the species distribution modeling. Variables enclosed in red boxes indicate pairs with high collinearity, defined as an absolute Pearson’s correlation coefficient |*r*| > 0.80. The nine variables retained for final modeling are those outside of the red boxes and not involved in strong collinearity.
